# Development and Validation of a Community Assessment Tool for Diverse Rural Family Caregivers

**DOI:** 10.1093/geroni/igaa057.319

**Published:** 2020-12-16

**Authors:** Michael McCarthy, Morgan Lee-Regalado Hustead, Rachel Bacon, Yolanda Evie Garcia, Dorothy Dunn, Heather Williamson, Julie Baldwin

**Affiliations:** 1 Northern Arizona University, Flagstaff, Arizona, United States; 2 Northern Arizona University, Flagstaff, United States

## Abstract

The aging of the United States population has led to an increase in the prevalence of Alzheimer’s Disease and Related Dementias (ADRD). Many individuals with ADRD receive care from family members and friends who, themselves, experience poor health outcomes. ADRD family caregivers from diverse backgrounds are frequently underserved by health and social service systems. However, research suggests that diverse caregivers possess culturally-based strengths that serve as protective factors against poor outcomes. This presentation reports findings from a study to develop and validate a community assessment of the needs, assets, and resources of rural Latinx and Native American ADRD family caregivers. Initial assessment items were based upon validated measures developed for diverse ADRD and comparable caregiver populations. A Delphi process was used to obtain ratings from a 9-member expert panel (ADRD policy makers, providers, and family caregivers) about the degree to which the assessment was: (1) inclusive of different cultural groups; (2) respectful of cultural values and norms; (3) comprehensive with respect to needs, assets, and resources, and; (4) relevant to the lived experiences of diverse rural caregivers. Experts also provided qualitative feedback about modifying phrases to make the assessment more relevant to diverse respondents, removing sections, and adding items and response options to better reflect rural life. After two formal rounds of review and multiple iterations, between 77% and 100% of experts agreed that all sections of the assessment met criteria. Findings highlight the benefits of a systematic process for developing and validating community assessments for diverse, rural populations.

